# Experience of CT diagnosis and management of primary renal Ewing’s sarcoma: A retrospective analysis of 6 cases and a literature review

**DOI:** 10.1097/MD.0000000000032189

**Published:** 2022-12-09

**Authors:** Xianwen Hu, Dandan Li, Jiong Cai

**Affiliations:** a Affiliated Hospital of Zunyi Medical University, Department of Nuclear Medicine, Zunyi, China; b Zunyi Hospital of Traditional Chinese Medicine, Department of Obstetrics, Zunyi, China.

**Keywords:** CT, Ewing’s sarcoma, kidney, primary neuroectodermal neoplasm, renal, renal

## Abstract

To present the clinical experience of primary renal Ewing’s sarcoma/primitive neuroectodermal tumors (rEWs/PNET) admitted to our hospital and systematically review the published literature. A retrospective analysis was performed on patients with pathologically confirmed renal EWs/PNET (rEWs) in our hospital, and the literature on rEWs published in PubMed and Embase databases before March 1, 2022 was searched for analysis. A total of 337 rEWs were included in the statistical analysis, including 6 cases of our patients and 331 cases published in the literature. The common clinical symptoms of rEWs are abdominal pain, hematuria, abdominal mass and so on. computed tomography (CT) plays an important role in the diagnosis of rEWs, and the typical manifestation is a large heterogeneous soft tissue density mass, with a specific “septum-like” enhancement in contrast-enhanced scan. The 2-year overall survival rate of rEWs was 48%, with a median survival time of 18 months. “Septum-like” enhancement on CT can be used as a relatively specific sign for the differential diagnosis of rEWs from Wilms tumor and neuroblastoma. The maximum diameter of the rEWs was usually greater than 10 cm, the clinical symptoms of weight loss, metastasis at diagnosis, tumor thrombogenesis of renal vein or/and inferior vena cava tumor, and the failure to undergo radical nephrectomy were the factors of poor prognosis. The incidence of primary rEWs is low and the prognosis is poor. Early diagnosis and radical nephrectomy combined with chemotherapy is the key to improve the prognosis of patients, and CT plays an important role in early diagnosis.

## 1. Introduction

Both Ewing’s sarcoma (EWs) and primary neuroectodermal tumors (PNET) share the same genetic and histological characteristics and are currently referred to collectively as EWs/PNET.^[1]^ EWs/PNET is a small round cell malignant tumor originating from neural crest cells and has the potential of multidirectional differentiation to neurons, glia and interstitial tissues, and is prone to local recurrence and distant metastasis. EWs/PNET is mostly found in bone and soft tissue, and primary renal EWs/PNET (rEWs) is rare clinically, accounting for less than 1% of renal tumor.^[2]^ Renal EWs is mostly single and usually originates from the renal medulla or renal pelvis, which is more common in young people. The median age of onset is about 30 years old, and the incidence of rEWs is higher in males than in females.^[3]^ As with other renal malignancies, the common clinical symptoms of rEWs are abdominal pain, hematuria and abdominal mass, with no specificity. Laboratory test results from rEWs were mostly unremarkable, but LDH and NSE levels have been reported to be above normal.^[4]^ Because of its highly invasive nature, rEWs is prone to cystic degeneration, necrosis, and intratumoral bleeding. The specificity of imaging has not yet been determined, and histopathological examination and molecular studies remain accurate methods for the diagnosis of rEWs.^[5,6]^ The rEWs is highly invasive and the disease progresses rapidly, so the patients often have lymph node, lung, bone or bone marrow metastasis at the time of diagnosis, with a poor prognosis.^[7,8]^ So far, most studies of primary rEWs have been based on case report(s). This present study retrospectively analyzed 6 cases of rEWs confirmed by pathology, immunohistochemistry and fluorescence in situ hybridization, and the published rEWs in the literature before March 1, 2022 was reviewed, as well as the clinical characteristics, computed tomography (CT) features, treatment and prognosis were summarized.

## 2. Methods

### 2.1. Study population

This was a retrospective analysis of primary rEWs confirmed by pathology, immunohistochemistry and fluorescence in situ hybridization as of March 1, 2022. The criteria for the inclusion of cases included the following: patients with detailed data such as gender, age, CT examination data, treatment methods, and patient prognosis, and confirmed by pathology and immunohistochemistry as primary Ewing sarcoma of the kidney.

English-language case reports and case series on rEWs were searched in PubMed, Embase and the Cochrane Library databases until March 1, 2022. The following retrieval strategy: (kidney OR renal) AND (Ewing sarcoma OR Ewing’s sarcoma OR primitive neuroectodermal tumor) were used for retrieval. Data such as gender, age, maximum tumor diameter, CT image characteristics, treatment methods, and follow-up outcomes of patients in the published studies were extracted for inclusion in statistical analyses. The entire process was carried out independently by 2 authors.

### 2.2. CT examinations

CT examinations were performed in all patients using 16-detector-row scanners (SIEMENS SOMATOM Sensation, Germany) from the top of the liver to the lower pole of the kidney. The enhanced scan was performed with iohexol (300mg I/ mL)1.5mL/kg, and a single phase injection at a rate of 3.0mL/s was performed with a high pressure syringe. Cortical phase images were collected 30 to 36 s after contrast medium injection, and parenchymal phase images were collected 60 to 70 s after contrast medium injection. All the 6 patients in this group received plain CT scan and enhanced examination. All the images were reviewed by 2 experienced imaging chief physicians to observe the location, shape (including size, edge), density (internal composition, uniformity, cystic degeneration, calcification, degree of enhancement, etc) and the relationship with the surrounding tissues and organs.

### 2.3. Statistical analysis

Statistical analyses were carried out with SPSS Statistics 25.0 (IBM Corporation, Armonk, NY) software packages. Median survival time (MST) and overall survival were calculated by using the Kaplan–Meier method. Overall survival -time was calculated from primary diagnosis to first event (death) or last follow-up. Univariate comparisons were estimated by the log-rank test. Multivariate analyses were carried out by Cox’s proportional hazard method. Chi-square or Fisher’s test was used to test proportions. *P* < .05 (2-sided) was considered statistically significant.

## 3. Results

### 3.1. Clinical features of patients with rEWs

According to the inclusion criteria, 6 patients who were previously treated in our hospital were included in this study, whose clinical data are presented in Table 1, including 4 males and 2 females, the age range from 13 years to 49 years old. All patients had a history of abdominal pain, one of whom also presented with fever and hematuria. At the time of diagnosis, all patients were localized except for 1 patient with bone and lymph node metastasis. All 6 patients underwent radical nephrectomy and chemotherapy. Follow-up times ranged from 3 to 78 months, during this process, 5 patients experienced progression and/or distant metastasis over a period of time ranging from 3 to 24 months.

**Table 1:** Clinical features of the patients with rEWs of present study.

Case	Age/Sex	History	Metastasis (diagnosis)	Recurrence	Treatment		Follow-up(mo)	status
					RN	Chemotherapy		
1	13/M	ABD pain	N	N	Y	VDC + i.e.,	25	Alive
2	18/F	Loin pain, hematuria, fever	N	Lung (3mo)	Y	VDC	4	Dead
3	41/M	ABD pain	N	Lung + liver	Y	CAV + i.e.,	8	Dead
4	49/F	Loin pain	LNs + bone	Progression	Y	CAV + i.e.,	6	Dead
5	31/M	Loin pain	N	LNs (2 mo)	Y	CAV	3	Dead
6	31/M	ABD pain	N	Lung (24 mo)	Y	CAV + i.e.,	78	Dead

M = male, F = female, Y = yes, N = not, ABD = abdomen, RN = radical nephrectomy, rEWs = renal EWs/PNET, VDC = (vincristine + daunorubicincy + cloadenosine phosphate), i.e. = (ifosfamide and etoposide), CAV = (cyclophosphamide + adriamycin + vincristine).

**Figure 1.**
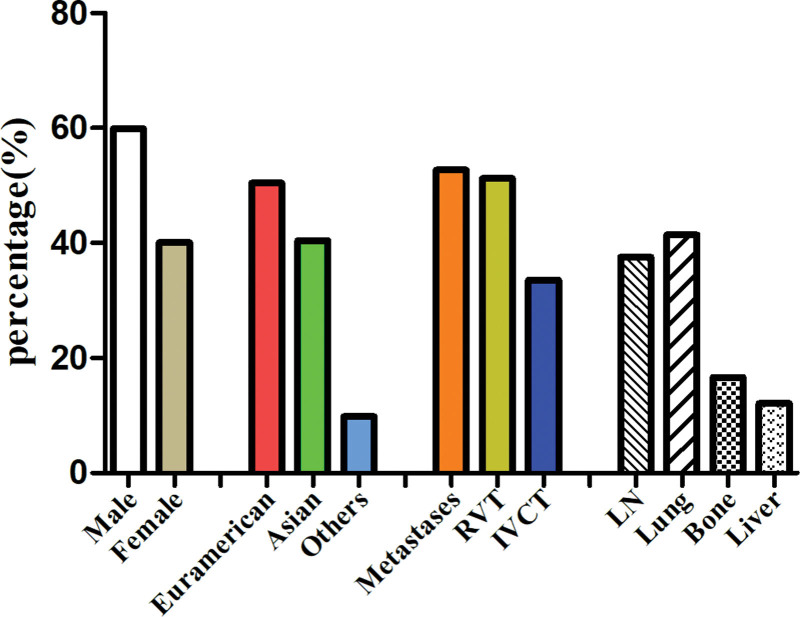
Percentage of clinical and characteristic distribution of renal Ewing’s sarcoma. RVT = Renal vein thrombosis; IVCT = Inferior vena cava thrombosis; LN = Lymph node.

**Figure 2.**
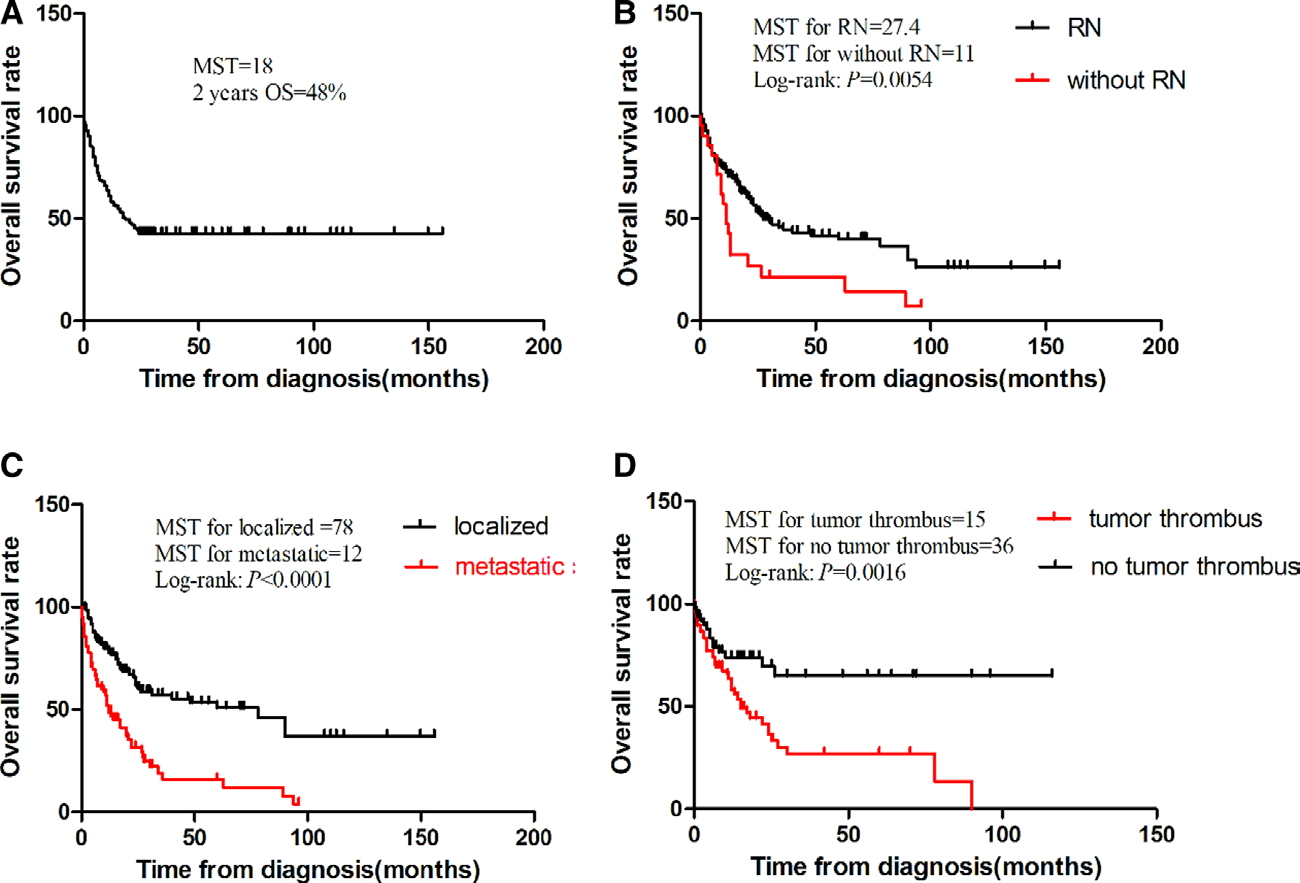
Kaplan–Meier overall survival (OS). (A). Two-year OS and median survival time (MST) in patients with renal Ewing sarcoma (N = 142); (B). OS according to whether radical nephrectomy (RN) was performed (N = 186); (C). OS according to disease stage at diagnosis (N = 190); (D). OS according to presence of tumor thrombus (N = 116).

After a systematic literature search, a total of 130 studies with 331 patients with rEWs were retrieved. Both our study results and findings from previously published research are summarized in Table 2. A total of 337 rEWs were included in the statistical analysis, including 6 in our present study and 331 in the literature published. Of the 337 patients with rEWs, 50.4% (136/337) were from Euramerican, 40.4% (136/337) were from Asia, and only 9.8% were from other regions. The clinical and characteristic percentage distribution of rEWs is shown in Figure 1. Gender data available for 307 patients in the study, with no significant difference between men and women (*P > .05*), including 184 males (59.9%) and 123 females (40.1%), the ratio of males to females is 3:2. The mean age at diagnosis was 30.7 years (data available for 237 patients), the median age was 30 years, and the age range was from 2 months to 78 years. The patients presented with clinical symptoms (data available for 237 patients) including abdominal or loin pain (167 cases,70.5%), hematuria (83 cases, 35.0%), abdominal mass (50 cases, 21.1%), fever (24 cases, 10.1%), and weight loss (22 cases, 9.3%), other rare symptoms include fatigue, edema, and acute hypertension. The maximum diameter of the tumor was statistically different between the group less than or equal to 10 cm and the group greater than 10 cm (*P < .05*), most of which had a maximum diameter greater than 10 cm at the time of diagnosis. Metastases occurred in 52.7% of patients at the time of diagnosis (157 of 298 patients), among which 37.6% (59 of 157 cases) had lymph node metastasis, 41.4% (65 of 157 cases) had lung metastasis, 16.6% (26 of 157 cases) had bone or bone marrow metastasis, 12.1% (19 of 157 cases) had liver metastasis, and other metastasis sites included pancreas, spleen, and peritoneum. Besides, of the 211 patients with complete data, 108 patients (51.2%) had renal vein thrombosis at the time of diagnosis, 71 patients (33.6%) had inferior vena cava thrombosis, and 7 patients (3.3%) had right atrium thrombosis. In addition to our 6 patients, 271 patients in the literature had detailed treatment information, 9.4% (26 of 277 patients) had only received radical nephrectomy, while most of the patients (70.4%, 195 of 277 patients) had also received radical nephrectomy and chemotherapy, and only a few patients just received chemotherapy (9.7%,27 of 277 patients) or chemotherapy plus radiotherapy (4.3%,12 of 277 patients). Follow-up data were available in 216 of 337 patients, and the mean follow-up time was 24.6 months, as well as the range was 0.2 to 156 months. Of these 216 patients, 125 patients (57.9%) occurred tumor recurrence or distant metastasis. The 2-year overall survival rate was 48%, with a MST of 18 months (Fig. 2A). The MST for patients undergoing radical nephrectomy was 27.4 months, and the median survival time for patients receiving radiotherapy alone, chemotherapy alone, or radiotherapy plus chemotherapy was 11 months, with statistically significant differences between the 2 (*P* = .0054, Fig. 2B). The MST of patients with localized tumor was 78 months at the time of diagnosis, and that of patients with metastatic disease was 12 months, with important statistical significance (*P* < .0001, Fig. 2C), and the MST for patients without tumor thrombus was 36 months compared with a MST of 15 in patients with tumor thrombus, also with statistically significant differences between them (*P* = .0016, Fig. 2D).

**Table 2:** Results and findings of rEWs both present study and literature published.

parameters	Variable	Present study	Literature	Total	*P*-value
Gender	Data available	6	301	307	.351
	Male	4 (66.7%)	180 (59.8%)	184 (59.9%)	
	female	2 (33.3%)	121 (40.2%)	123 (40.1%)	
Age at diagnosis (yr)	Data available	6	231	237	
	Mean (range)	30.5 (13–49)	30.7 (0.2–78)	30.7 (0.2–78)	
	Median	31	30	30	
Size(cm)	Data available	6	196	202	**.016**
	MD < 10	4 (66.7%)	79 (40.3%)	83 (41.1%)	
	MD ≥ 10	2 (23.3%)	117 (59.7%)	119 (58.9%)	
Presenting symptoms	Data available	6	231	237	
	ABD or loin pain	6 (100%)	161 (69.7%)	167 (70.5%)	
	hematuria	1 (16.7%)	82 (35.5%)	83 (35.0%)	
	ABD mass	0 (0.0%)	50 (21.6%)	50 (21.1%)	
	fever	0 (0.0%)	24 (10.4%)	24 (10.1%)	
	Weight loss	0 (0.0%)	22 (9.5%)	22 (9.3%)	
Metastasis at diagnosis	Data available	6	292	298	.564
	Yes	1 (16.7%)	156 (53.4%)	157 (52.7%)	
	No	5 (83.3%)	136 (46.6%)	141 (47.3%)	
Metastasis sites	Data available	1	156	157	
	lymphonodus	1 (100%)	58 (37.2%)	59 (37.6%)	
	lung	0 (0%)	65 (41.7%)	65 (41.4%)	
	Bone/bone marrow	1 (100%)	25 (16.0%)	26 (16.6%)	
	liver	0 (0%)	19 (12.2%)	19 (12.1%)	
	other	0 (0%)	14 (9.0%)	14 (9.0%)	
Treatment	Data available	6	271	277	
	RN	0 (0%)	26 (9.6%)	26 (9.4%)	
	RN + C	6 (100%)	189 (69.7%)	195 (70.4%)	
	RN + C + R	0 (0%)	16 (5.9%)	16 (5.8%)	
	C	0 (0%)	27 (10.0%)	27 (9.7%)	
	C + R	0 (0%)	12 (4.4%)	12 (4.3%)	
	R	0 (0%)	1 (0.4%)	1 (0.4%)	
Follow-up	Data available	6	210	216	
	alive	1 (16.7%)	109 (51.9%)	110 (50.9%)	
	dead	5 (83.3%)	101 (48.1%)	106 (49.1%)	

ABD = abdomen, MD = maximum diameter, R = radiotherapy, rEWs = renal EWs/PNET, C = chemotherapy, RN = radical nephrectomy.

### 3.2. CT findings of rEWs

**Figure 3.**
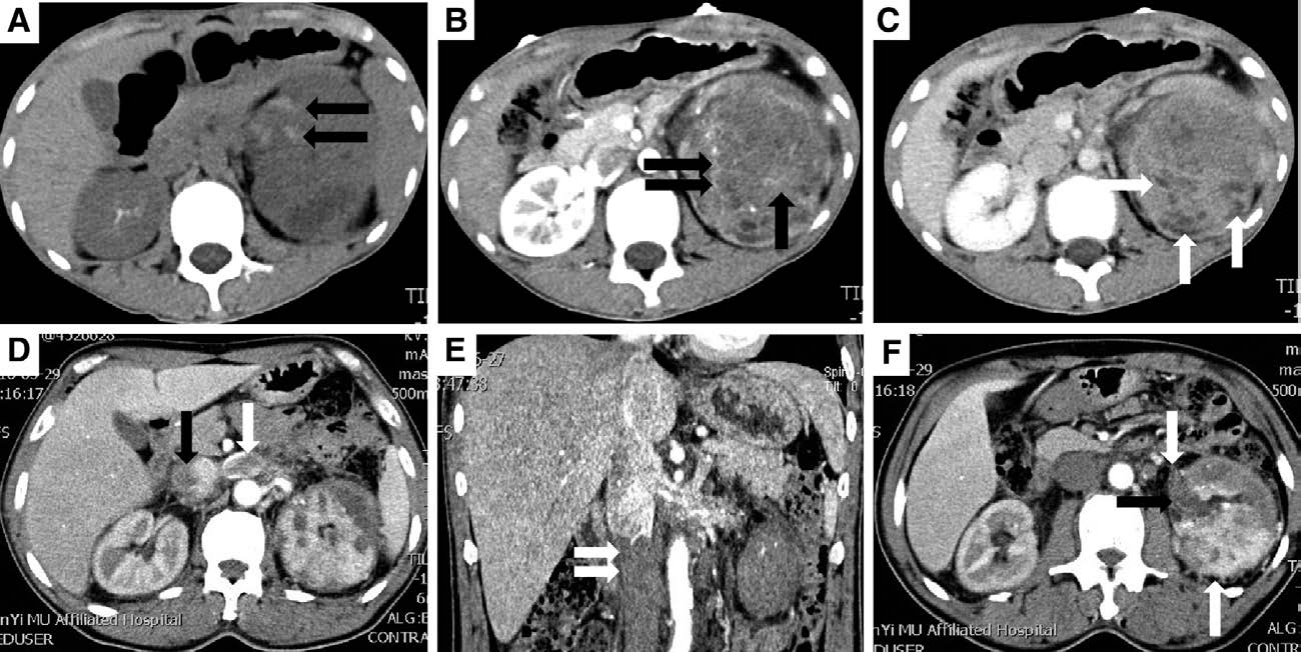
(A). A 12-year-old male patient was hospitalized due to abdominal pain and accidental hematuria, CT revealed a large soft tissue mass with inhomogeneous density in the left renal region, and nodules and patchy high-density hemorrhagic shadows (black arrows); (B). In the cortical stage of contrast-enhanced CT, the lesion was slightly inhomogeneous in enhancement, and the septum-like enhancement shadow was visible in it (black arrows); (C). Contrast-enhanced CT in the nephrographic phase of the lesion continued to non-uniform enhancement, the tumor surrounding a number of small nodular cystic lesions with no enhancement (black arrows); (D). An 18-year-old woman was admitted to hospital due to abdominal pain. Contrast-enhanced CT showed an inhomogeneous density soft tissue mass shadow in the left kidney, filling defects in the renal vein (white arrow) and inferior vena cava (black arrow), suggesting tumor thrombosis; (E). The coronal position of multiplanar reconstruction clearly shows the thrombus of inferior vena cava (white arrows); (F). Contrast enhanced CT showed tumor invasion of renal sinus (black arrow) and perirenal fat (white arrows). CT = computed tomography.

CT findings of 6 cases of rEWs in the present study are shown in Table 3. Of the 6 rEWs, 4 were in the left kidney and 2 were in the right kidney, and the maximum diameter of the tumor ranged from 5.0 cm to 25.6 cm. All cases had inhomogeneous density due to cystic necrosis (6 cases) and hemorrhage (3 cases), and only 1 case had high density calcification in the tumor parenchyma. At the process of dynamic enhanced scan, all cases presented progressive enhancement, among which 5 cases presented mild enhancement in the cortical phase and moderate enhancement in the nephrographic phase, and 1 case presented moderate enhancement in the cortical phase and significant enhancement in the nephrographic phase. In 5 of the 6 cases, renal sinuses were involved and perirenal fat or fascia was involved in 3 cases, and 2 had both renal venous thrombosis and inferior vena cava thrombosis. Typical CT images of rEws are shown in Figure 3.

**Table 3:** CT features of the patients with rEWs of present study.

No	Side	MD (cm)	Density	Cyst/Necrosis	HE	Ca	Enhancement degree/type			Invasion		Tumor embolus	
							CP	NP	SE	PF	RS	RV	IVC
1	L	12.8	IH	+	+	−	Mild	Moderate	+	+	+	−	−
2	L	8.5	IH	+	−	−	Mild	Moderate	+	−	+	+	+
3	R	25.6	IH	+	+	+	Moderate	Marked	+	+	+	−	−
4	L	7.2	IH	+	+	−	Mild	Moderate	+	−	+	−	−
5	L	5.0	IH	+	−	−	Mild	Moderate	+	−	−	−	−
6	R	7.0	IH	+	−	−	Mild	Moderate	−	+	+	+	+

L = left, R = right, IH = inhomogeneous, MD = maximum diameter, HE = hemorrhage, Ca = calcification, CP = cortical phase, CT = computed tomography, NP = Nephrographic phase, SE = septum-like enhancement, PF = perirenal fat, rEWs = renal EWs/PNET, RS = renal sinus, RV = renal vein, IVC = inferior vena cava.

Both our CT results and findings from previously published research are summarized in Table 4. Of the 252 available patients’ data, the tumor location was almost equally distributed, with 128 on the left and 123 on the right. More than half of the tumors had a maximum diameter greater than 10.0 cm (119 of 202 patients, 58.9%). The unenhanced CT scan showed that virtually all tumors (142 of 147 patients, 96.6%) had inhomogeneous density, with 141 (95.9%) patients showing low-density cystic necrosis in the tumor parenchyma, 48 patients (32.7%) with intratumoral hemorrhage, and only 17 patients (11.6%) with high-density calcification in the tumor parenchyma. In the contrast-enhanced cortical phase, 77.3% of the tumors showed mild enhancement, 22.7% showed moderate enhancement, and in the parenchymal phase, 38.1% showed sustained mild enhancement, 49.5% showed moderate enhancement, and 22.7% showed significant enhancement, and 87 of the 97 patients (89.7%) showed septum-like enhancement. Of the 106 patients’ available CT data, 50 tumors (47.2%) invaded perirenal fat or fascia, and 72 tumors (67.9%) invaded renal sinuses.

**Table 4:** CT results and findings of rEWs both present study and literature published.

Parameters	Variable	Our patients	Literature	Total
Unenhanced CT features	Data available	6	141	147
	Inhomogeneous	6 (100%)	136 (96.5%)	142 (96.6%)
	Cyst/Necrosis	6 (100%)	135 (95.7%)	141 (95.9%)
	Hemorrhage	3 (50%)	45 (31.9%)	48 (32.7%)
	Calcification	1 (16.7%)	16 (11.3%)	17 (11.6%)
CECT (Renal cortical phase)	Data available	6	91	97
	Mild	5 (83.3%)	70 (76.9%)	75 (77.3%)
	Moderate	1 (16.7%)	21 (23.1%)	22 (22.7%)
	Evident	0 (0.0%)	0 (0.0%)	0 (0.0%)
CECT (Nephrographic phase)	Data available	6	91	97
	Mild	0 (0.0%)	37 (40.7%)	37 (38.1%)
	Moderate	5 (83.3%)	43 (47.2%)	48 (49.5%)
	Evident	1 (16.7%)	11 (12.1%)	12 (12.4%)
	Septum-like enhancement	5 (83.3%)	82 (90.1%)	87 (89.7%)
Invasion	Data available	6	100	106
	Perirenal fat/fascia	3 (50.0%)	47 (47.0%)	50 (47.2%)
	Renal sinus	5 (83.3%)	67 (67.0%)	72 (67.9%)
Tumor embolus	Data available	6	205	211
	Renal veins	2 (33.3%)	106 (51.7%)	108 (51.2%)
	Inferior vena cava	2 (33.3%)	69 (33.7%)	71 (33.6%)
	Right atrial	0 (0.0%)	7 (3.4%)	7 (3.3%)

Y = yes, N = not, L = left, R = right, MD = maximum diameter, CT = computed tomography, CECT = Contrast-enhanced computed tomography, rEWs = renal EWs/PNET.

## 4. Discussion

It is known from the literature that EWs/PNET is most common in bone, trunk, pelvis, retroperitoneum, soft tissue parts of limbs and the central nervous system while less common in the kidney.^[9]^ In addition to the 6 rEWs cases we are studying, to our knowledge, 331 cases of rEWs have been reported in the published literature, and we compared and integrated the data from the current study with published data. The pathogenesis of rEWs is relatively complex, and some literature suggested that it may be related to the chromosomal translocation of EWSR1, but it has not been confirmed yet.^[10,11]^ The clinical manifestations of rEWs are similar to other renal malignant tumors, including lumbago or abdominal pain, hematuria and fever, and nonspecific manifestations such as abdominal mass and weight loss as the disease progresses, in addition, several cases of rEWs were reported in the literature with acute hypertension as the first symptom, which may be related to the secretion of endocrine active substances by tumor.^[12–15]^

**Figure 4.**
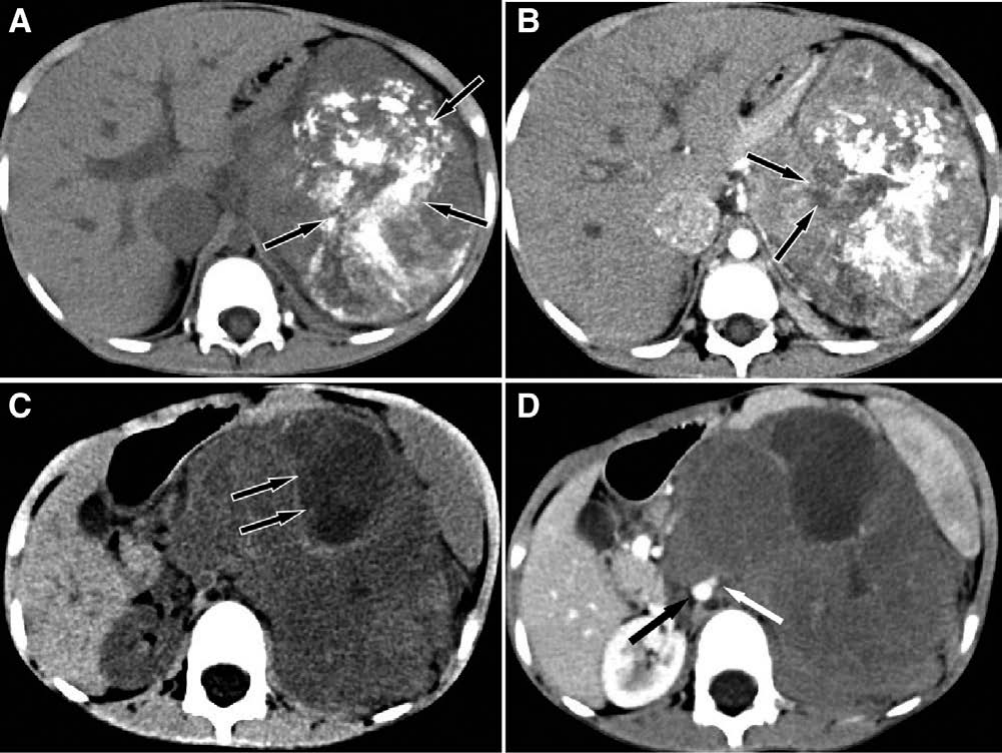
A 9-year-old girl with pathologically confirmed Wilms tumor, and CT showed a huge soft tissue mass in the left retroperitoneum with multiple calcifications (A, black arrows). Contrast-enhanced scans showed that the mass was heterogeneously enhanced, with low-density and non-enhancing necrotic areas (B, black arrows). A 5-year-old male patient with pathologically confirmed renal neuroblastoma, and CT showed a huge, superficially foliated, iso-soft-tissue density mass in the left kidney, and a large low-density cystic area was seen in the left kidney (C, black arrows). Contrast-enhanced scan showed mild enhancement of the lesion, and the mass grew around the abdominal aorta (D, black arrow) and left renal artery (white arrow). CT = computed tomography.

Imaging examination plays an important role in the detection of renal space-occupying lesions, especially CT, which can clearly show the origin, tumor morphology, size and invasion scope of lesions. From the CT data of our present study and literature published, it can be known that the CT signs of rEWs has a certain relative specificity: it is usually presented as a single, large, fuzzy boundary and irregular soft tissue mass in the kidney. EWs/PNET is highly invasive and prone to necrosis, cystic degeneration, and intratumoral bleeding, resulting in inhomogeneous density. In the present study and literature published, only several cases had relatively uniform lesion density. The density of the solid portion of the mass is equal to or slightly less than that of the renal parenchyma, the density of necrotic and cystic components is low. Calcification occurred in tumor tissues of a small proportion of rEWs patients. Because of the aggressiveness of the tumor, 1 to 3rd of patients have an inferior vena cava or renal vein thrombosis at diagnosis. The enhancement degree of the substantive part of rEWs in dynamic enhanced scanning was varied, most of which presented mild or moderate enhancement at the renal cortical phase, and moderate or evident enhancement at the renal parenchymal phase. Some studies^[16,17]^ believed that multiple irregular septum-like structure and delayed enhancement in lesions are characteristic manifestations of rEWs and can be used to differentiate from other renal tumors. In younger patients, rEWs needs to be differentiated from nephroblastoma, the most common tumor in children. The CT image of the latter also shows a large mass with equal or slightly lower density, necrosis, cystic change and hemorrhage are also common, and calcification can be seen in some lesions (as sown in Fig. 4A, B). On contrast-enhanced scans, Wilms tumors almost always show heterogeneous enhancement.^[18]^ Moreover, rEWs also need to be differentiated from renal neuroblastoma, another rare tumor of the kidney. A typical renal neuroblastoma is a huge lobulated soft tissue mass, which is easy to surround and bury renal blood vessels. It is also prone to necrosis, cystic degeneration, and hemorrhage, but it has a high probability of calcification, which usually presents as sandy or massive calcification.^[19]^ On contrast-enhanced scans, renal neuroblastoma does not show “septum-like” enhancement in rEWs, which is helpful for its identification (as sown in Fig. 4C, D). Renal clear cell carcinoma (RCC) is less common in childhood, and tumors are usually smaller in greatest dimension than sarcomas. Most RCC were significantly enhanced at the cortical phase of contrast-enhanced CT, and the parenchymal phase and delayed scanning of the lesion rapidly reduce the enhancement degree, presenting a typical “fast in and out,” while the enhancement degree of rEWs in cortical and parenchymal phase was far lower than that of RCC.^[20]^

At present, the diagnosis rEWs is mainly based on pathology and immunohistochemistry. Under the microscope, the tumor is mainly composed of primitive small round cells with dense staining and relatively consistent morphology, which is easy to see mitotic image. Immunohistochemistry showed that tumor cells consistently and diffusely expressed CD99, and expressed Vimentin, Syn and NSE to varying degrees, while CgA a.nd S-100 were often negative.^[21,22]^

There is no unified treatment guideline for rEWs, and the treatment principles of other renal malignancies or osseous EWs are mainly referred to at present.^[23]^ Radical nephrectomy (RN) is the main treatment method for rEWs, and adjuvant therapy such as chemotherapy and radiotherapy can be decided after surgery according to the patient’s condition.^[24–26]^ A study suggested that the recurrence rate after RN is still high even if the tumor does not metastasize at the early stage, and chemotherapy adjuvant therapy should still be routinely performed.^[27]^ Of our enrolled patients (both our present study and literature published), 85.6% (237 of 277 patients) underwent RN or RN combined with chemotherapy/radiotherapy, and only 40 patients just received chemotherapy and/or radiotherapy due to multiple distant metastases. The commonly used chemotherapy drugs of EWs/PENT include vincristine, cyclophosphamide and doxorubicin, etc, which can be changed or not according to the patient’s tolerance.^[28–30]^ In a study by Gaetano Bacci et al, the effect of radiotherapy on EWs is not clear and should be considered only in terminal patients or when perirenal fascia is involved.^[31]^ According to the data in Table 2 and the survival curve of Figure 2, the gender, age, tumor location and whether the patient has abdominal pain, hematuria, abdominal mass or fever have no statistical significance for the prognosis of the patient. However, tumor size, weight loss as the first clinical symptom, metastasis, tumor thrombosis, and radical nephrectomy received for patients were independent prognostic factors. To be specific, patients with tumors with a maximum diameter of more than 10 cm, with symptoms of weight loss at the time of visit, and with tumor thrombosis, metastatic disease at the time of diagnosis, and with failure to undergo radical nephrectomy had a lower average survival rate. Therefore, early accurate diagnosis is of great significance for the prognosis of patients.

The limitations of our study were mainly due to the rarity of rEWs, which resulted in a small number of enrolled cases. Most of the data in the statistical analysis were obtained through literature retrieval, and the reliability of the data is unknown. Furthermore, we only integrated the CT features of rEWs in the present study with those in the published literature, so a prospective study with relatively large sample size was designed to confirm the diagnostic value of CT in rEWs that needs to be considered in future work.

## 5. Conclusion

To sum up, athough rEWs is relatively rare clinically, the CT signs such as tumor size is relatively large, most of which are associated with cystic necrosis and hemorrhage, and mild-to-moderate enhancement and “septum-like” enhancement presented by contrast-enhanced CT scan are relatively specific. Radical nephrectomy combined with chemotherapy is currently the preferred treatment for rEWs, even so, the rEWs has a poor prognosis, with a 2-year disease-free survival rate of less than 50%. Early accurate diagnosis is an important factor for good prognosis of patients, and CT is an effective and important means to obtain early diagnostic information.

## Acknowledgments

The authors would like to express their sincere gratitude to colleagues in the Department of Pathology and Medical Records of the Affiliated Hospital of Zunyi Medical University for providing the clinical data and pathological results of the patients.

## Author contributions

**Conceptualization:** Xianwen Hu.

**Data curation:** Dandan Li.

**Formal analysis:** Dandan Li.

**Funding acquisition:** Jiong Cai.

**Methodology:** Xianwen Hu.

**Software:** Dandan Li.

**Writing – original draft:** Xianwen Hu.

**Writing – review & editing:** Jiong Cai.
